# Combined Evaluation of IGF−1 and IGFBP−3 as an Index of Efficacy and Safety in Growth Hormone Treated Patients

**DOI:** 10.4274/jcrpe.v1i5.240

**Published:** 2009-08-05

**Authors:** Zeynep Şıklar, Gönül Öcal, Merih Berberoğlu, Pelin Bilir

**Affiliations:** 1 Ankara University School of Medicine, Department of Pediatric Endocrinology, Ankara, Turkey; +90 312 215 82 54zeynepsklr@hotmail.comOrman Fidanlık Lojmanları 23/4, 06560 Ankara, Turkey

**Keywords:** IGF−1, IGFBP−3. growth hormone therapy

## Abstract

**Objective**: Measurement of serum insulin−like growth factor−1 (IGF−1) and IGF binding protein−3 (IGFBP−3) levels has been recommended as a useful index for monitoring of growth hormone (GH) therapy in GH deficient children. In this study we aimed to evaluate IGF−1/IGFBP−3 molar ratio during GH treatment as an index of safety and efficacy.

**Methods**: Serum IGF−1 and IGFBP−3 levels and molar ratio of IGF−1/IGFBP−3 were evaluated in 50 GH deficienct children, during 3 years of GH therapy and these parameters were compared with the growth response.

**Results**: All patients completed the first year, 38 the second year and 26 the third year of therapy. Although 15 patients in the first year, 5 patients in the second year, and 5 patients in the third year had high IGF−1 SDS values, height increments were similar in the low IGF−1 group and in the normal or high IGF−1 level groups.  Molar ratios were also not statistically different between the groups. Molar ratio of IGF−1/IGFBP−3 seemed to be more reliable in evaluating the efficacy than basal IGF−1 level.

**Conclusions**: Evaluation of the molar ratio of IGF−1/IGFBP−3 may be recommended as a tool to monitor  GH treatment and it may be possible to individualize GH treatment accordingly.

**Conflict of interest:**None declared.

## INTRODUCTION

Guidelines about growth hormone (GH) dosing continue to evolve and new data on the efficacy and safety of GH treatment keep emerging. Recently, concerns have been raised regarding excessive GH exposure in patients receiving GH therapy manifested as elevated insulin like growth factor (IGF)−1 levels. Age−adjusted IGF−1 is generally accepted as the biochemical marker to monitor GH treatment. It is known that serum (IGF)−1 values are above the age−related reference range in several patients receiving GH therapy. Whether GH dose should be altered or therapy discontinued accordingly is a controversial issue (1, 2, 3). IGF binding protein (IGFBP)−3 is also a GH dependent parameter and is affected by GH dose in a similar way. IGF−1 makes up a complex with IGFBP−3 and acid labile subunit. The molar ratio of IGF−1 and IGFBP−3 reflects the availability of the free form of IGF−1 which is effective for growth stimulation. Using the molar ratio was suggested as a safety index of GH treatment (4).  

The aim of this study was to evaluate IGF−1 and IGFBP−3 levels and molar ratio in patients on GH treatment and to compare these parameters with the growth response of the patients.

## METHODS

In this longitudinal study, 50 GH deficient children were evaluated during a 3−year period. The diagnosis of GH deficiency was made on the basis of clinical and laboratory data. Children with short stature (height below −3 SDS for chronological age), subnormal height velocity (HV, below 25th centile for age), delayed bone age (more than 2 years) and subnormal GH secretion (GH max <10 ng/ml) in two provocative tests (L−Dopa and insulin tolerance tests) were diagnosed as GH deficient. Within the GH deficient group, 35 patients were diagnosed as isolated complete GH deficiency (peak GH response to stimulation tests <5 ng/ml) and 15 patients had isolated partial GH deficiency (peak GH response to stimulation tests >5ng/ml to <10 ng/ml). Patients with multiple growth hormone deficiency were not included in the study. Recombinant human GH therapy was given to patients in a dose of 0.2 mg/kg/week. Growth response of GH treated patients was evaluated as delta height SDS (the difference in height SDS between basal value and that found in the consecutive year). 

IGF−1 was determined in the serum by an immunoradiometric assay [IRMA, Diagnostic Systems Laboratories (DSL−5600), Webster, Tex., USA]. IGFBP−3 was measured by IRMA using DSL−6600. IGF−1 and IGFBP−3 were expressed as SDS (5).  

For calculating the “molar ratio” of IGF−1 to IGFBP−3, the following molecular masses were used: IGF−1  7.5 kD and IGFBP−3 42kD (6).

The statistical analyses were done using the SPSS package programme. Kruskal Wallis, Pearson’s correlation and Mann−Whitney U tests were used.

## RESULTS

All patients completed the first year of therapy, 38 patients the second year and 26 patients the third year of therapy. Yearly height gain, expressed as delta height SDS, and parameters related to IGF−1 and IGFBP−3 are shown on Table 1. Fifteen patients in the first year, 5 patients in the second year and 5 patients in the third year were found to have high IGF−1 SDS values for age and pubertal status.  

**Fig. 1:**
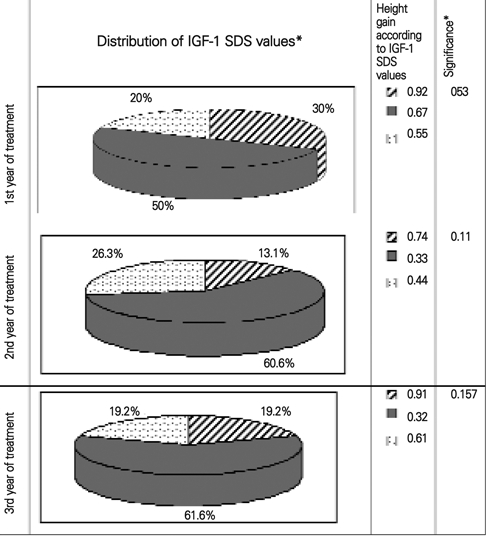
Comparison of height gain and IGF−1 SDS values on GH therapy

There was no correlation between IGF−1 SDS and delta height SDS over the first year (p=0.53). No extra height gain was observed in patients with IGF−1 SDS values higher than +2 SDS. Molar ratio was also not different in patients with high IGF−1 SDS as compared to patients with normal or low IGF−1 SDS (Fig. 1). 

During three  years of treatment height gain was not correlated to IGF−1 SDS.  (Correlation coefficients were:  r=−0.082, and p=0.618 for the first year of treatment, r=−0.173, and p=0.35 for the second year of treatment, and r=−0.105, and p=0.67 for the third year of treatment, respectively). Although 15 children (30%) in the first year, 5 patients (13.1%) in the second year, and 5 patients (19.2%) in the third year of therapy showed high IGF−1 levels (>+2SDS), IGF−1/IGFBP−3 molar ratio of these children was not higher than the others.

**Table 1:**
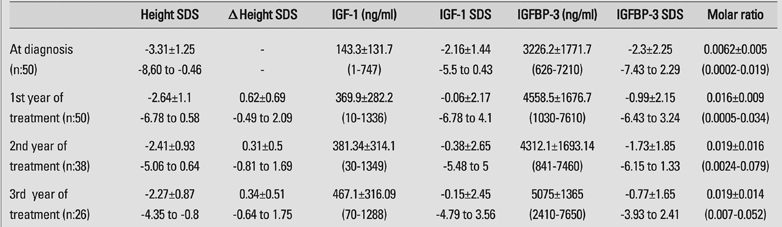
Height increments expressed as delta (Δ) height SDS and parameters related to IGF−1, IGFBP−3 and molar ratio in GH deficient children receiving GH treatment

## DISCUSSION

GH therapy normalizes the subnormal IGF−1 levels in GH deficient patients (7). However, IGF−1 levels may increase to supranormal values and this increase leads to concerns about potential hazardous effect of IGF−1. IGF−1 is a mitogenic polypeptide that stimulates cell proliferation and suppresses cellular apoptotic pathways to facilitate cell growth (8). An increased level of IGF−1 receptor expression is present in some malignant tumors (9).  Several case−control studies have demonstrated a strong association between circulating IGF−1 concentrations and the relative risk of specific cancers (10, 11, 12). Prostate, breast, colorectal, lung cancers are the most studied cancer types, and a link has been shown to exist between IGF−1 levels and increased risk of carcinogenesis (11, 12, 13, 14, 15, 16). 

IGFBP−3 is the most abundant binding protein of IGF−1 in the serum, and is an important component of the natural physiological regulation of IGF (9).  IGFBP−3 appears to have a protective effect, inhibiting the mitogenic effect of IGF−1 on cell proliferation by lowering the amount of the free bioactive form of IGF−1. IGFBP−3 also facilitates apoptosis, possibly independent of its effect on IGF−1 (8).  Because a long−term sustained elevated serum IGF−1 concentration may dispose to carcinoma, it is recommended that measurements of IGF−1 and IGFBP−3 should be done regularly in GH treated subjects (17). 

GH therapy increases both IGF−1 and IGFBP−3 levels and helps to maintain a normal IGF−1/IGFBP−3 ratio, thereby having a compensatory effect  for high IGF−1 levels (4). While monitoring GH therapy, not only IGF−1 levels but also  IGFBP−3 levels may be important, and calculating IGF−1/IGFBP−3 molar ratio may give a crude estimate of  the free IGF−1 level. 

There was a significant increase in height SDS over the first year of therapy in our patients, declining over the consecutive years. Delta height SDS was +0.62 at the end of the first year, +0.31, in the second year, and +0.34 in the third year. After onset of therapy, the molar ratio was almost tripled, and stayed at a similar level (approximately 0.019) during the three−year follow−up. We could not find any correlation between delta height SDS and mean IGF−1 SDS for each year. Patients with low and high IGF−1 levels showed similar height increments during the 3 years of treatment. This similar height gain can be explained in patients having a similar molar ratio at all stages, probably reflecting a similar degree of free IGF−1 in all groups. This equilibrium between IGF−1 and IGFBP−3 may have caused the same level of bioavailibility of free IGF−1 at tissue level. Unfortunately, we did not measure free IGF−1 levels in our patients and therefore could not analyze free IGF−1 together with molar ratio. It should also be noted that free IGF−1 levels may not be similar even if molar ratios are the same and that molar ratios may be a crude reflection of free IGF (18). However, it may be concluded that higher dose of GH treatment would not be necessary in patients with low IGF−1 levels unless the molar ratio is also low.  

In conclusion, concomitant elevation of IGFBP−3 and IGF−1 leads to a similar molar ratio in GH treated patients. Evaluation of IGF−1 together with  IGFPB−3 and calculating the molar ratio may be useful  for monitoring the GH effect and for individualizing the therapy to enhance efficacy and safety.
